# Normalization of low-density microarray using external spike-in controls: analysis of macrophage cell lines expression profile

**DOI:** 10.1186/1471-2164-8-17

**Published:** 2007-01-17

**Authors:** Paolo Fardin, Stefano Moretti, Barbara Biasotti, Annamaria Ricciardi, Stefano Bonassi, Luigi Varesio

**Affiliations:** 1 Laboratory of Molecular Biology, G. Gaslini Institute, Genoa, Italy; 2 Unit of Molecular Epidemiology, National Cancer Research Institute, Genoa, Italy

## Abstract

**Background:**

The normalization of DNA microarrays allows comparison among samples by adjusting for individual hybridization intensities. The approaches most commonly used are global normalization methods that are based on the expression of all genes on the slide and on the modulation of a small proportion of genes. Alternative approaches must be developed for microarrays where the proportion of modulated genes and their distribution are unknown and they may be biased towards up- or down-modulated trends.

**Results:**

The aim of the work is to study the use of spike-in controls to normalize low-density microarrays. Our test-array was designed to analyze gene modulation in response to hypoxia (a condition of low oxygen tension) in a macrophage cell line. RNA was extracted from controls and cells exposed to hypoxia, mixed with spike RNA, labeled and hybridized to our test-array. We used eight bacterial RNAs as source of spikes. The test-array contained the oligonucleotides specific for 178 mouse genes and those specific for the eight spikes. We assessed the quality of the spike signals, the reproducibility of the results and, in general, the nature of the variability. The small values of the coefficients of variation revealed high reproducibility of our platform either in replicated spots or in technical replicates. We demonstrated that the spike-in system was suitable for normalizing our platform and determining the threshold for discriminating the hypoxia modulated genes. We assessed the application of the spike-in normalization method to microarrays in which the distribution of the expression values was symmetric or asymmetric. We found that this system is accurate, reproducible and comparable to other normalization methods when the distribution of the expression values is symmetric. In contrast, we found that the use of the spike-in normalization method is superior and necessary when the distribution of the gene expression is asymmetric and biased towards up-regulated genes.

**Conclusion:**

We demonstrate that spike-in controls based normalization is a reliable and reproducible method that has the major advantage to be applicable also to biased platform where the distribution of the up- and down-regulated genes is asymmetric as it may occur in diagnostic chips.

## Background

Studies on gene expression rely heavily on DNA microarray technology [1]. In a typical microarray experiment, the two RNA samples to be compared are reverse transcribed in cDNA, labeled using two different fluorophores and then hybridized simultaneously to the glass slide to measure the relative gene expression level [2]. Essential to the analysis of microarray data is the normalization process, which allows comparison among samples by adjusting for individual hybridization intensities. There are many approaches to normalize expression levels and the most commonly used, referred to as global normalization methods, apply to experiments in which most of the genes are equally expressed in both channels [3]. The global normalization approach is based on the use of the majority of genes on the slide to normalize microarray experiments and a constant adjustment is used to force the distribution of signal ratios to have the same measure of central tendency, e.g., the same median. These methods can be applied when the elements spotted on the array are representative of a random and large number of genes [4] and when there is symmetry in the frequency of the up/down-regulated genes [5]. Alternative approaches have to be developed when the majority of the genes represented on the array are coordinately up- or down-modulated as in the case of diagnostic chips [3,6]. Diagnostic chips are designed as low-density microarrays containing a number of selected genes expected to be concomitantly up- or down-regulated in response to given signals, drugs, or pathological conditions. The advantage of low-density over high-density platforms is the competitiveness in price and the flexibility of design.

We propose the use of external reference RNAs (also known as spike-in controls or spikes) to normalize the data of low-density microarray. Spike RNAs show no sequence similarity to the genome of the studied species and they are added in defined amounts to experimental RNA samples before labeling. The oligonucleotides specific for the spike RNAs are spotted onto the slide. The use of spikes allows not only data normalization but also the evaluation of several parameters of the platform quality, including the sensitivity and specificity of the microarray experiments, the accuracy and reproducibility of the measurements and the assessment of technical variability introduced by labeling procedure, hybridization and image scanning [7,8].

Our laboratory is involved in the study of the cellular response to hypoxia, a condition of low oxygen tension that characterizes many pathological situations [9]. Hypoxia occurs in cardiovascular, hematological, and pulmonary disorders, inflammatory processes, and fibrosis [10]. Areas of low oxygen concentration are present in solid tumors and are known to contribute to tumor growth, metastasis, and resistance to radio and chemotherapy [11]. We and others have applied microarray technology to define the profile of gene expression associated to hypoxia utilizing the Affymetrix GeneChip [12] and we are in the process of designing low-density microarrays that will identify the hypoxia-inducible genes in tumor specimens, and may serve as prognostic indicators of the aggressiveness of the disease and of the sensitivity to therapy. A prerequisite for the development of such tool is a correct normalization procedure and a sound analysis of the data that does not require preexisting information on the expected pattern of results and that will be suitable even if the majority of the genes is modulated or the distribution of up- or down-regulated genes is asymmetric.

In this study, we demonstrate that a composite loess normalization [13,14] based on spike-in controls is the proper way to deal with low-density microarray platforms that applies also to extreme distribution of the data when the global normalization approaches will generate erroneous results.

## Results and discussion

### Experimental planning

We describe a normalization/validation procedure of a low-density array (test-array) based on spike-in controls (spike RNA). The spike RNA consists of eight, commercially available, purified bacterial mRNAs with no similarity to the mouse genome (Table 1). The corresponding 50 mers were spotted on the test-array which contains a total of 48 replicates of each spike printed by 4 different pins and distributed on the slide as described in the Methods section. The experiments were aimed at evaluating spike signals when variable amounts of spike RNA are added to experimental RNA and hybridized to the test-array. Experimental RNA was purified from the murine macrophage cell line ANA-1 [15] that was cultured in a normoxic or hypoxic environment for 18 hrs in order to define the hypoxia inducible genes. Experimental RNA from normoxic and hypoxic macrophages was spiked with the eight different spike RNAs. The samples were labeled with red and green fluorophores, mixed, hybridized to the test-array and tested for fluorescence signals. The oligonucleotides spotted on the test-array represented 178 mouse genes induced- or inhibited- by hypoxia or by other stimuli and those specific for the spike RNAs. Rough data were filtered on the bases of the signal and only signals with more than 50% of the pixels falling two standard deviations over the local background were considered for the analysis. This filtering procedure, described by Bahler *et al. *[16], discarded unreliable data coming from weak signals, caused by nonspecific hybridization, and retained those in which at least half of the spot was significantly brighter than the background. Whenever a spot did not pass this criterion for one channel but in the other channel had more than 95% of the pixels satisfying the inclusion criteria, the spot was included in the analysis. The reason for this procedure was the effort of including in the analysis genes weakly expressed in one experimental condition but with reliable expression in the other [16].

### Spikes' signal

Initial experiments were performed to characterize the signals of the spike RNA in the biological setting. Experimental RNA from control and hypoxia treated macrophages were mixed with equal amounts of each spike RNA generating an expected ratio of 1 in the spike signal. The experiments were designed in such a way that each pin printed quadruplicate subarrays containing the entire set of eight oligonucleotides corresponding to the spikes and one fourth of the oligonucleotides specific for the experimental genes.

The first issue that we addressed was the reproducibility of the signal of the spike RNA. We calculated the coefficient of variation (CV) for each spike on the 48 replicates present on each individual test-array and we averaged the CV and standard deviations (SD) of seven independent test-arrays analysed (Table 2). We found that the CV ranged from 5.15% to 8.10% depending on the spike, indicating low variation and a good level of reproducibility of the results similar to what described for other platforms [16,17]. This conclusion was supported by the analysis of the standard deviations that were quite homogeneous and ranged from 0.05 and 0.11.

Analysis of variance (ANOVA) was used to estimate the contribution of each potential source of error to the overall variability. For this purpose, several pairs of experimental RNA from control or hypoxic macrophages were mixed with increasing amounts of the eight spike RNAs (10, 250, 750, 1000 pg) each of which generated an expected ratio of 1. The ANOVA explored the association between the log ratio of all spikes and the potential sources of variance including spike characteristics (genomic sequence, base composition, length of the mRNA, interaction with the other RNA in the sample, etc.), mRNA spike concentration, position on the array (spot size, tips, slide homogeneity, printer accuracy, etc.), and interaction term between the spike characteristics and spike concentration. The results are shown in Table 3 in the "Before normalization" panel. We found that the major portion of the variability could be attributed to the spike concentration (48.3%) followed by the interaction term (29.7%), and the spike characteristics (17.8%). The position on the array did not seem to contribute significantly to the variability of the system (0.4%), in contrast to what observed in other platforms [5].

The difference between the observed ratios of the various spikes and the expected ratio of 1 was rather small but occasionally some outlier spikes were far from 1. We explored the possibility to reduce the variability eliminating outlier values. Outliers were defined as those spikes showing either great variability among replicates or ratios far from 1. We developed a filtering procedure to keep the spikes that showed low variability, defined as CV less than 10%[18], and a mean ratio falling 1 ± two standard deviations to exclude values in the extreme 5% of the distribution. Table 4 shows the results of one experiment in which we utilized the data of the seven independent microarray experiments used to calculate the reproducibility shown in Table 2. The mean ratios and standard deviations of the eight spikes within each experiment were calculated and the outliers were removed using the above criteria. The results demonstrated that some spikes were very reproducible and did not generate any outlier. In some instances one or two outliers out of seven replicates (Table 4) were detected and excluded from the analysis of the data. After the removal of the outliers a significant decrease in the CV was observed demonstrating the value of the filtering procedure.

In summary, these results clearly show that the information provided by spikes' signal is reproducible, characterized by low variability (that can be further reduced by filtering our outliers values) and suitable to normalize the results of our test-array.

### Normalization of the spikes' signal

The relative fluorescence intensity between channels must be normalized to adjust for systematic biases such as differences in RNA levels, dye incorporation and detection efficiencies [13]. The dye balance may vary with spot intensity and with spatial position on the array. Loess and print-tip loess normalization are among the most commonly used methods to remove such variability although the application of this algorithm is not free from potential risk on the interpretation of the results [5,14]. We utilized, as a routine, the loess normalization procedure, which does not take into consideration the tip-to-tip variability because, under the present experimental conditions, the ANOVA showed that the contribution of the position on the array to the total variability were negligible.

**Figure 1:**
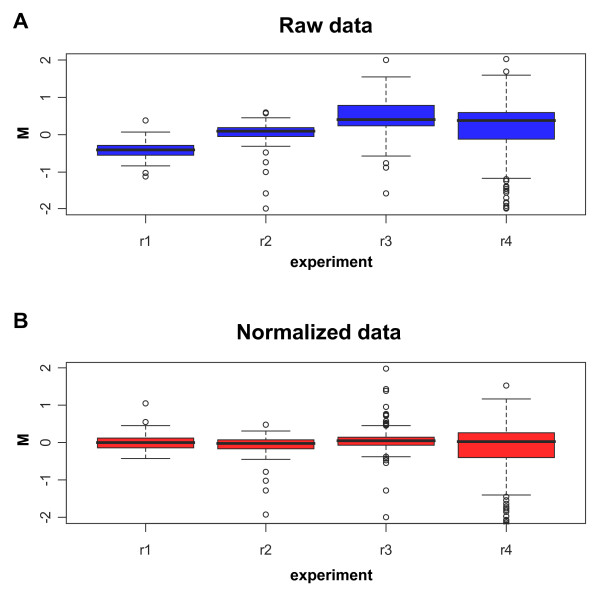
Box plots of the expression log-ratios (M-values) of spike genes of four replicates (r1–r4). Each box corresponds to one array where the amount of spike RNA on both channels is 250 pg. **(A) **Box plots before normalization (i.e. only background subtracted M-values of raw data). **(B) **Box plots after normalization (i.e. loess normalized M-values).

We applied a composite loess normalization to the spikes' signal [14], which corrects the expression log-ratios for intensity-based trends by subtracting from each expression log-ratio the corresponding value of the loess curve. The loess curve is constructed exclusively on the spike-in controls expression data and it is not biased by experimental genes values [13,14]. Being R and G the background-corrected red and green intensities for each spot respectively, the expression log-ratio (M-value) corresponding to a generic spot is M = log_2_R - log_2_G, whereas the log-intensity (A-value) of each spot is defined as A = (log_2_R+log_2_G)/2. The effect of normalization on the distribution of the ratios of the spikes in four independent measurements is shown in the box-plots in Figure 1. Before normalization (Figure 1A) the values are dispersed around the theoretical value of zero, but following loess normalization (Figure 1B) the spreading was clearly diminished providing further evidence of the need for normalization in comparing different experiments.

To define the effects of normalization on the variability the spikes' signal data were analyzed by ANOVA (Table 3, "After normalization") and compared to those obtained in the absence of normalization ("Before normalization"). When dissecting such variability into its components we found a major decrease for the spike concentration (from 48.3% to 1%) indicating that any spike concentration within the range from 10 to 1000 pg could be used, including low amounts of spike with a substantial saving of reagents.

The normalization did not affect the variability associated to the array position, and most variability is now explained by the spike characteristics (40.4%) or by their interaction with the spike concentration (47.4%). The little change in the R squared between the two ANOVA models (from 0.958 to 0.887) is due to the change in the relationship between the components and the interaction term and it does not affect the reliability of the model.

**Figure 2:**
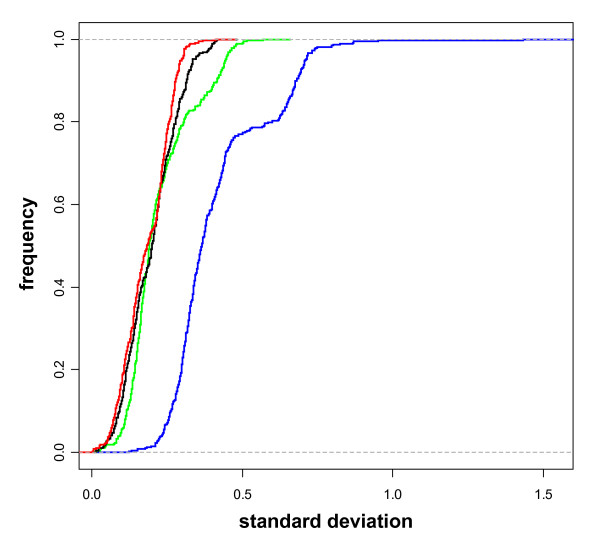
Empirical Cumulative Distributions (ECDs) of standard deviation of the M-values for spikes on loess-normalized data (red line), loess normalized and scaled via the *quantile *method (green line), loess normalized and scaled via the *scale *method (black line) and raw data (blue line) on seven technical replicates. The graph shows the frequency (Y-axis) of the standard deviation of the M-values (X-axis). As a term for comparison, we provide the values of the median standard deviations corresponding to the ECDs: red = 0.18, green = 0.19, black = 0.20, blue = 0.36.

Comparison among different experimental arrays may require a scalarization of the data following normalization to adjust for dyes unbalance. We assessed the possibility of a further improvement of the data consequent to the spike signals scalarization. Figure 2 shows the empirical cumulative distributions (ECDs) of the standard deviations for M-values on four technical replicates in which data were not normalized (blue line), normalized but not scaled (red line), normalized and scaled through the *quantile *method (green line) [19], or normalized and scaled through the *scale *method (black line) [3,5,14]. The shift of the blue line confirms the importance of normalization of the results. The red, green and black lines are almost superimposed showing that the scaling process does not provide any practical improvement in terms of standard deviation of M-values. The small deviations of the black and green line curves from the red one suggest that the noise introduced by the scaling may be more detrimental than a small difference in scale. We conclude that spike signal normalization is a necessary step for subsequent use as internal reference whereas scaling is not required.

### Analysis of the experimental platform using the spikes

**Figure 3:**
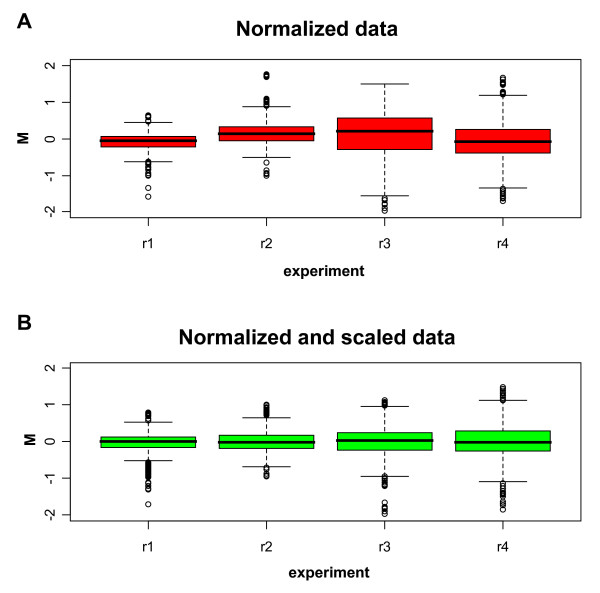
Box plots of the M-values on four technical replicates (r1–r4) for normalized experimental genes. The amount of spike RNA on both channels is 250 pg. **(A) **Box plots before scaling. **(B) **Box plots after the *quantile *scale method.

**Figure 4:**
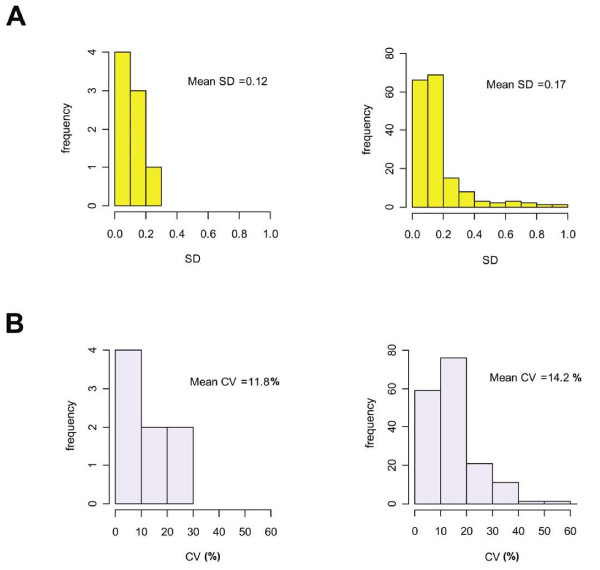
Array data reproducibility. **(A) **The histograms represent the distribution of the standard deviations (SD) of M-values of technical repeats for the spikes (left side) and for the experimental genes (right side). The mean SDs of the repeated measurements of each group are shown. **(B) **The histograms represent the distribution of the coefficients of variation (CV) [(SD of M-values × 100)/mean of M-values] of technical repeats for the spikes (left side) and for the experimental genes (right side). The mean CVs of all repeated measurements of each group are shown.

Having identified experimental conditions for optimal analysis of the spikes' signal, we studied the experimental RNA expression values following spike-in based normalization. The loess normalization based on spike-in controls corrects the whole set of expression data constructing a loess curve on spikes log-ratios. The experimental RNA log-ratios are corrected for intensity-based trends subtracting from each expression log-ratio the corresponding value of the loess curve [14]. The normalized results of four replicate experiments in which we added 250 pg of spike RNA to the experimental RNA are represented as box plots in Figure 3A. A variation of the spread and of the alignment among replicate experiments is evident suggesting the need for scaling to compare the results. After scaling with the *quantile *method [19] (Figure 3B) a good alignment and a similar spread can be demonstrated indicating that scaling is important for the experimental data although not needed for the spikes themselves as previously discussed. The variability of the experimental data following normalization and scaling is measured by calculating the standard deviations (SD) and the coefficient of variations (CV) of the signal from spikes and experimental genes among replicate arrays (technical repeats) (Figure 4). The variability of the technical repeats measured on spikes' signal (Figure 4, left side) was very similar to that calculated on experimental genes (Figure 4, right side) indicating a high degree of reproducibility of the platform.

**Figure 5:**
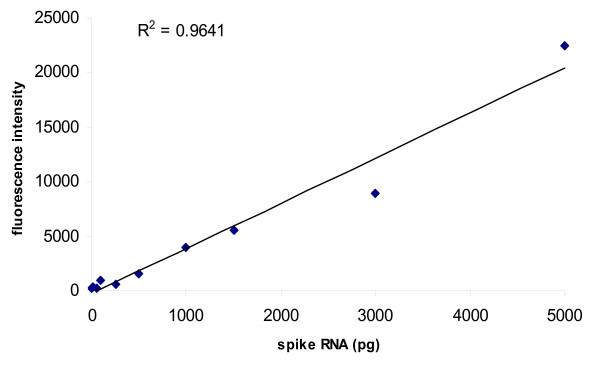
Detection limit of spiked RNA samples. 15 μg of total RNA were spiked with spike RNAs at increasing concentrations: 5 pg, 10 pg, 50 pg, 100 pg, 250 pg, 500 pg, 1000 pg, 1500 pg, 3000 pg and 5000 pg. The mean fluorescence intensity was plotted as a function of transcript concentration. The black line represents the linear regression calculated by the least squared method (R-squared = 0.9641).

Quantitative spiking experiments were carried out to determine the sensitivity of the system. Increasing amounts of spike (ranging from 5 to 5000 pg) were added into a sample of 15 μg of experimental RNA (Figure 5). We found that the signal readout was linear over the entire range of concentrations. The detection limit was at a dilution of 1:150,000 (w/w), which corresponds approximately to 5 pg of specific mRNA.

**Figure 6:**
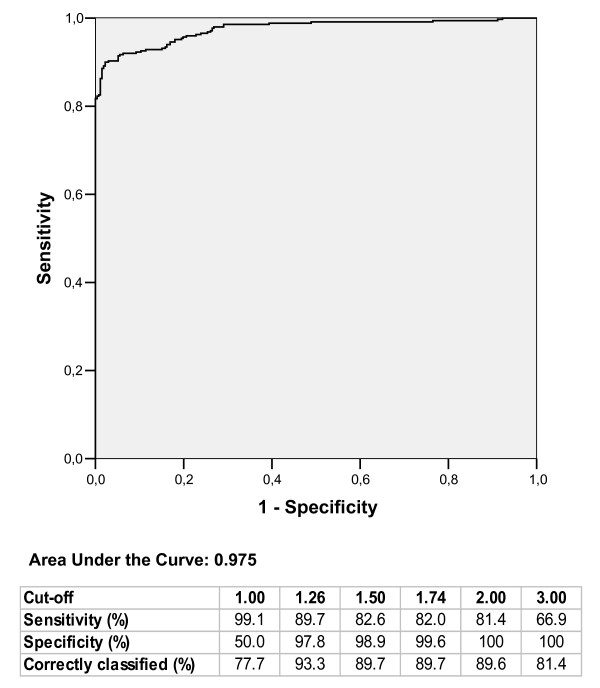
ROC curve for experiments leaded with known concentration of 8 spike genes. The area under ROC curve gives an estimate of the test accuracy. In the table are reported sensitivity, specificity, and the proportion of correctly classified genes corresponding to the expected ratios of 1, 1.5, 2, 3 and to the optimal thresholds of 1.26 and 1.74 provided by the ROC analysis.

**Figure 7:**
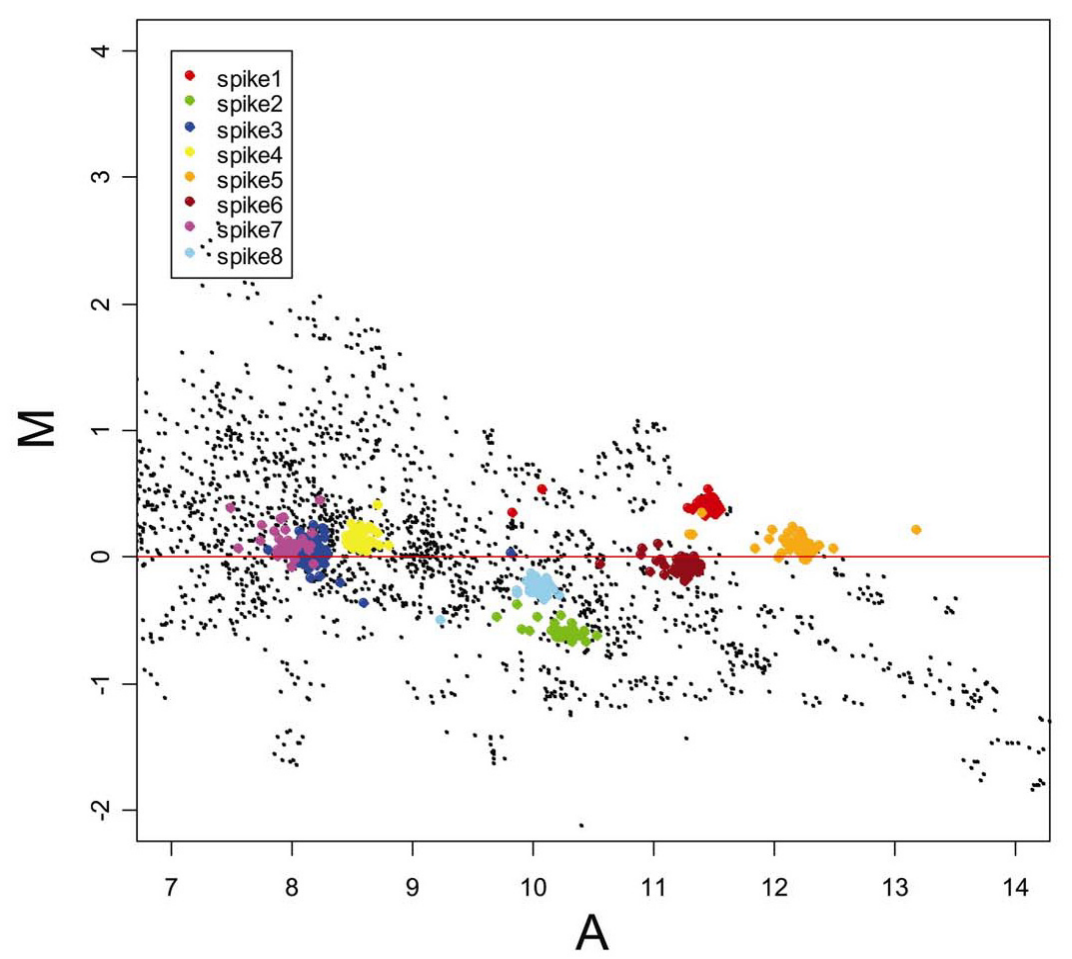
MA-plots of the experimental genes (black dots) and of the spikes (represented with eight different colors) after loess normalization. The spikes are distributed around M = 0 and span about 75% of the range of log-intensity A.

To define the theoretical cut-off ratio identifying regulated genes, we calculated the ROC curve [20] on the values obtained in experiments in which spikes were added at pre-defined amounts to obtain ratios of 1 (500/500 pg), 1.5 (750/500 pg), 2 (1000/500 pg) and 3 (1500/500 pg). Dye swap was performed to generate reverse ratios. The results are shown in Figure 6 where the true positive rate (sensitivity) is plotted against the false positive rate (1-specificity) for the different thresholds applied. We defined the genes with ratios = 1.5, 2, 3 as positive and genes with ratio = 1 as negative. ROC curve analysis was performed comparing the distribution of positive versus negative genes. The ROC curve indicates that 1.26 is the best cut-off ratio to discriminate regulated genes from non-regulated genes, with a sensitivity (true regulated genes) of 89.7%, a specificity (true non-regulated genes) of 97.8% and a rate of genes correctly classified of 93.3%. According to our experimental system, 1.26 is the ratio corresponding to the point on the ROC curve nearest to the left-hand corner, representing the highest theoretical value of accuracy (probability of correct classification = 100%). The ratio of 1.74 resulted in 100% of probability to classify true regulated genes since above this ratio the false positive rate is equal to zero. We conclude that genes showing ratios above 1.74 are definitively up-regulated and below 1.26 are definitively non-regulated. The choice of thresholds at intermediate levels will be dictated by the nature of the experiment and the required stringency that can be deduced from the ROC table. Efficient normalization using the loess curve requires that the platform manifests specific characteristics in terms of spike properties and number of replicates. The first requirement is that the intensities of the signals of the various spikes span across the range of intensities of the experimental data [19]. The relationship between our experimental data and the eight spikes used is shown in Figure 7. The spike-in controls have a range of log-intensities spanning between 8 and 13, which comprises 75% of the 178 expression data. Such differences of log-intensity among spikes are caused by intrinsic characteristics including base composition, length of the mRNA and interaction with other RNA molecules, and they are unpredictable and must be verified for each set of spikes. In our situation, the spikes were suitable for normalization without further adjustments. Modification of the spike concentration and/or the number of spikes used must be considered in the event of a partial coverage of the range of intensities of the experimental data by the spikes' signal.

A second consideration is the strength of the loess curve with respect to the number of replicates. We calculated the minimum number of replicates needed to have a robust loess procedure. The robustness was calculated on the basis of reproducibility of the normalized data considering an increasing number of replicates and we set as a threshold for our system a CV of 10%. In our platform we have 8 spikes each replicated 48 times in each slide for a total of 384 replicates. We applied a Monte Carlo resampling procedure aimed at sampling 100 times the 384 replicates collecting randomly each time a fixed number of replicates (n < 384). Each sample was used for normalization of the array, the mean expression value of the experimental data was computed and the CVs based on each sample were calculated (data not shown). We found that the CV was less than 10% when the number of replicates was equal or greater that 200 and we concluded that a minimum of 200 replicates is needed for an accurate normalization of the platform. In our platform we spotted 48 replicates per spike and, theoretically, five spikes would be sufficient to reach the threshold number of 200 replicates. However, this is the minimum number required and five spikes would not allow, for example, the elimination of the outliers. On the bases of our limited experience, we propose that a platform that uses the spike-in normalization system, as that described here, must adhere to the requirement of a minimum of 200 replicates among all spikes and a set of spikes representative of the range of expression values of the experimental data. In general, the optimal number of spikes/replicates to be used will have to be calculated considering the range of log-intensities covered by the spikes and the reproducibility of the normalized data with an increasing number of replicates.

### Normalization and scaling of the experimental platform

**Figure 8:**
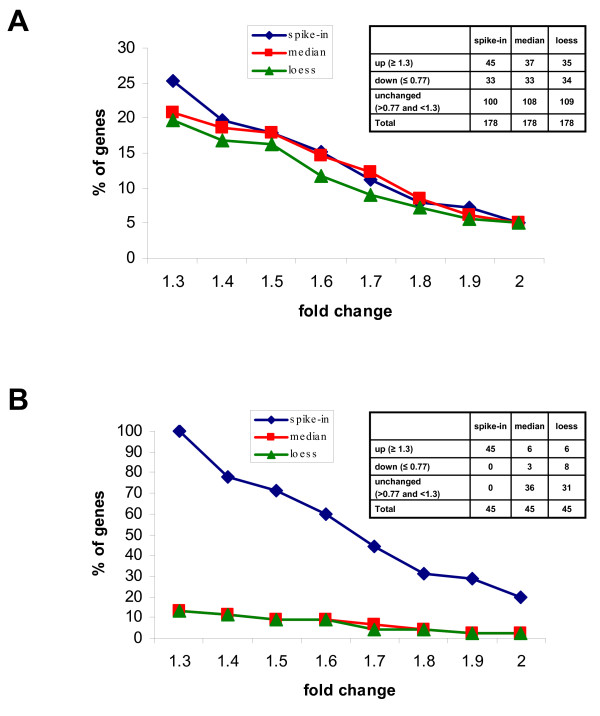
Effect of different normalization procedures on the detection of modulated genes. RNAs from control or hypoxia -treated macrophage cell line were mixed with the spikes and hybridized to our test-array. Data were filtered (including the outliers removal), normalized with spike-in method (blue line), with the median (red line) or with the loess based on all genes (green line) and scaled. The graphs plot the percentage of genes (Y-axis) having a value equal or greater than that indicated in the X-axis. **(A) **The analysis was carried on the entire test-array comprising 178 genes symmetrically distributed in up-regulated, down-regulated and not modulated. **(B) **The analysis was carried on 45 genes having a fold change equal or greater than 1.3 and representing an asymmetric distribution.

One purpose for developing this spike-in controls platform is the possibility of applying this methodology to normalize microarrays with an asymmetric representation of modulated genes. We modelled this situation in the analysis of the changes of gene expression in macrophages following exposure to hypoxia. RNAs from control or hypoxia-treated macrophage cell line were mixed with the spikes and hybridized to our test-array. The 178 gene expression results (Figure 8) were normalized using the method described here that applied the loess algorithm based on the spikes only (spike-in, blue line) or using the loess applied to all genes (loess, green line) or the median (median, red line) algorithm applied to all experimental data without including the spikes' values. We used the fold change of 1.3 as the threshold value to identify modulated genes as derived from the ROC curve analysis. We found 25% of up-regulated, 19% of down-regulated and 56% of unchanged genes in response to hypoxia (Figure 8A) when the data were normalized with the spike-based normalization. Similar results were obtained when the median normalization algorithm and the loess based on all genes were used. Furthermore, we plotted the frequency of genes with increasing fold change values. Similar curves were obtained using the three methods of normalization. These results demonstrated that the spike-based normalization is interchangeable with global normalization algorithms under condition in which the representation of modulated and not modulated genes is symmetric such as that shown in Figure 8A. The use of the frequency of genes with the given fold change is a read out of practical interesting that, however, is not suitable for measuring the relative performance of the normalization systems, based on assessment of relative biases and variances, that is thoroughly discussed in Park *et al. *[21]. Depending on the platform and on the aim of the analysis some normalization may perform better than others, but in our system with our read out we failed to appreciate significant differences. However, different results were obtained when we considered a case limit in which the microarray contained only up-regulated genes. We modelled this scenario by considering only the 45 genes up-regulated identified in Figure 8A. The distribution of modulated genes on the bases of the three normalization methods is shown in table of Figure 8B. The spike-based normalization identified 100% of the inducible genes as expected. In contrast, the median normalization algorithm identified only 13% inducible genes because it assumes a non-existing normal distribution of the data. The majority of the genes were classified as not changed. The loess normalization based on all genes generated analogous results. Similar conclusions were drawn when we plotted the frequency of genes with increasing fold change. The spike-based normalization originated a curve of frequency that had a similar slope as that observed in Figure 8A indicating that this normalization can be applied irrespectively to the symmetry of modulated genes on the array. In contrast, the curve generated with the median normalization algorithm and the loess based on all genes failed to identify up-regulated genes indicating that these methods can not be applied to an asymmetric distribution of genes such as that depicted in Figure 8B. In conclusion, the spike system has a broader applicability and is suitable for the analysis of asymmetric arrays because it normalizes the results according to an external reference independent from the experimental RNA and, therefore, not affected by the symmetry of the array.

The genes that are up-regulated in macrophages by hypoxia with a probability of 100% are listed in Table 5. 14 out of 17 genes are known to be up-regulated by hypoxia or they have been validated by independent assays including microarray with other platforms. In contrast, three genes were not previously associated with the hypoxic response (F2r, Foxg1, Ifi204) and are currently under investigation. These results confirm that we could detect hypoxia inducible genes and that our platform is suitable for testing hypoxia diagnostic chips to be applied to inflammatory diseases or cancer.

## Conclusion

We describe the use of spike-in external control to normalize a low-density microarray. The spikes are an external reference that allows data normalization independently from the expression of the experimental RNA particularly suitable for situation in which there is asymmetric distribution of modulated genes. This approach does not rely upon the low-density property of the array and, theoretically, it can be applied to high-density arrays. We can not exclude that the source of the experimental RNA may affect the spike performance and some evaluation of the parameters described here may be needed in different experimental conditions. We demonstrate that the application of loess normalization to the spikes' signal decreases significantly the major source of variability. Furthermore we introduce a criterion for the removal of the outliers that is quite useful to further reduce the system variability.

The choice of normalization method is critical for a correct interpretation of the results when the distribution of the expression values in not symmetrical and/or the number of spotted genes is limited. In fact, we show that median normalization method or loess normalization based on all genes spotted are unable to cope with situations in which only up-regulated genes are present on the array. Unfortunately, the nature of the distribution of gene expression is generally unknown and normalization methods that are independent from this variable are desirable. We demonstrate that the spike-in method is as effective as other global normalization methods in dealing with symmetric distribution of the expression values. More important, we show that it can be successfully applied also to situation in which the distribution of the expression values is highly asymmetric. We conclude that the spike-in system is a method of choice for arrays with a potentially biased distribution. A situation in which there is the potential for an asymmetric distribution is represented by low-density diagnostic chip where the choice of the genes spotted may be deliberately biased towards those that are up-regulated in a given pathological condition.

There is no reason to believe that the spike-in normalization method could not be suitable for high-density microarrays. The only possible technical limitation is the inclusion of spike probes in commercially available chips. However high-density microarrays are characterized by a symmetric distribution and commonly used normalization approaches are equally effective or possibly superior as the intensity-dependant normalization procedure. A thorough comparison among normalization methods has been published [21].

We describe the use of eight spikes for an accurate normalization of the platform and in our experience this conditions allows an accurate normalization in every experiment. On the basis of a Monte Carlo resampling procedure we determined that theoretical minimum number of five spikes each replicated 48 times is needed for the normalization of our platform. However, such number has severe limitations including the impossibility of excluding the outliers. In general, the number of spikes to be used in different applications may vary depending on the stringency of the criteria used, on the desired variability threshold and on the robustness of the platform and it may have to be recalibrated when using other sources of RNA.

Our experimental system consists of RNA from cell line cultured in normoxic condition or exposed to hypoxia, a condition of low oxygen tension that characterizes several pathological conditions. Very often, arbitrary expression cut-offs are set to discriminate between genes that are modulated or not changed. We describe here the successful use of the ROC curves to assess objectively the threshold to identify hypoxia-modulated genes. The expression data obtained with our platform are consistent with previous information generated in our laboratory using other platforms and with the data in the literature relative to the response of macrophages to hypoxia confirming that we could detected hypoxia inducible genes and that our platform is suitable for supporting hypoxia diagnostic chips.

In summary, we present an accurate description and characterization of a normalization procedure of a low-density microarray based on spike in external controls that has the potential of a broad applicability to different types of arrays including those in which there is an asymmetric distribution of the up/down-regulated genes.

## Methods

### Microarray fabrication

C6-amino-linker oligonucleotides (50 nucleotides in length) were obtained from MWG oligoset (MWG-Biotech AG, Ebersberg, Germany), and spike-in controls oligonucleotides were purchased from Ambion ArrayControl Spot (Ambion Inc., Austin TX). Oligonucleotides were printed on e-Surf Activated Slides (Life Line Lab S.r.l., Italy) with a SpottingArray 24 (PerkinElmer, Wellesley, MA) using 4 Stealth Micro Spotting Pins (Telechem International, Inc. Sunnyvale, CA) in 150 mM phosphate buffer pH 8,5 at 40% humidity. E-surf activated slides are obtained by adsorption on glass of a hydrophilic polymer containing N, Nacryloyloxysuccinimide (NAS). Oligonucleotides were printed at a final concentration of 10 pmol/μl. The coupling reaction was performed o/n in a saturated NaCl solution chamber with a 75% relative humidity. All oligonucleotides were printed in quadruplicates over 4 subarrays with a 2 × 2 print head. Spike-in controls oligonucleotides, negative control and buffer were printed in quadruplicates onto each subarray. The scheme is repeated 3 times on the entire slide surface resulting in 12 replicates for each gene element and 48 replicates for each control element.

### RNA preparation

ANA-1 cell line [15] was cultured and maintained at 37°C in a humidified incubator containing 20% O_2_, 5% CO_2 _and 75% N_2_. For hypoxic conditions, cells were incubated in a humidified anaerobic workstation incubator (BUG BOX, Ruskinn, UK) flushed with a mixture of 94% N2, 5% CO2 and 1%O2. Total RNA was extracted from ANA-1 cells grown under normoxic or hypoxic conditions for 18 hours, using Trizol (Invitrogen Life technologies, Irvine, CA) according to the manufacturer's protocol. The physical quality control of RNA integrity was carried out by electrophoresis using Agilent Bioanalyzer 2100 (Agilent Technologies, Waldbronn, Germany) and quantified by NanoDrop (NanoDrop Technologies, Wilmington, Delaware USA). Spike-in controls RNA were purchased from Ambion ArrayControl RNA Spikes. The RNA Spikes are a set of 8 purified RNA transcripts with sequence homology to the corresponding ArrayControl Spot. The ArrayControl sequences were selected from Escherichia coli genes that show no sequence similarity to mammalian genomes.

### Sample labeling and microarray hybridization

15 μg of total RNA were converted in either Cy3- or Cy5-labeled cDNA probe using the Superscript indirect cDNA labelling kit (Invitrogen Life technologies, Irvine, CA). Spike RNA were added in appropriately diluted 2 μl mixture to total RNA and to oligodT primer, RNase-free water was used to bring the volume to 18 μl, and the reaction was denatured at 70° for 5 min and then chilled on ice. Amminoallyl-modified cDNA was generated in the presence of 5× first-strand buffer, 0.1 M DTT, dNTP mix (including amino-modified nucleotides), RNaseOUT™ (40 U/μl), SuperScript™ III RT (400 U/μl) in a final volume of 30 μl at 46°C for 3 hours. RNA template was hydrolyzed by the addition of 15 μl of 1 N NaOH followed by heating at 70°C for 10 min. Reactions were neutralized with 15 μl of 1 N HCl, and cDNA was purified on S.N.A.P. columns according to the manufacturer's instructions followed by ethanol precipitation. cDNA was lyophilized to dryness and resuspended in 5 μl of 2× coupling buffer. NHS ester of Cy3 or Cy5 dye (Amersham Pharmacia, GE Healthcare Little Chalfont, UK) in DMSO (dye from one tube was dissolved in 5 μl of DMSO) were added and reactions were incubated at room temperature in the dark for 1 h. Coupling reactions were quenched by the addition of 20 μl of 3 M sodium acetate pH 5.2, and unincorporated dye was removed using S.N.A.P. columns. The combined Cy3 and Cy5 probes were dried down in a speed-vac and then dissolved in 6 μl of RNase-free water. 10 μg of Cot-1 DNA, 10 μg of poly(A) and 4 μg of yeast tRNA were added, the mixture was denatured at 95°C for 3 min and then cooled down on ice for 1 min. 35% formamide, 3.5× SSC, 0.3% SDS and 2.5× Denhardt's were added to a final volume of 90 μl. Slides were blocked in an appropriate blocking solution, 100 mM ethanolamine, 0.2 M Tris, pH 9.0, at 50°C for 20 min and then washed in 4× SSC, 0.1% SDS for 20 min. Blocked slides were pre-hybridized at 42°C for 45 minutes with a pre-hybridization mixture (35% formamide, 4× SSC, 0.5% SDS, 2.5× Denhardt's, 20 ng/μl Salmon Sperm DNA) in the HS 400 hybridization station (Tecan Austria GmbH, Salzburg, Austria). Hybridizations were carried out at 42°C for 16 h automatically agitated every 5 min, followed by washing in (3 min each): 2× SSC and 0.1% SDS, 1× SSC and 0.5× SSC at room temperature.

### Data acquisition, normalization and analysis

Arrays were scanned using a GenePix 4000B dual-color confocal laser scanner (Axon Instruments, Union City, CA) at 10-micron resolution. Images were processed, and signals from spotted arrays were quantitated using GenePix Pro 5.1 software (Axon Instruments). Array images that did not pass minimal quality control were discarded (median signal-to-background >3; median signal-to noise >3; mean of median background signal <200). Technically imperfect spots were removed either automatically by the GenePix software or through manual investigation of the array images. Such spots were flagged as 'absent' in the GenePix results files and they were not included in the analysis. To discard data from weak signals, spots with <50% of pixels >2 SD above median local background signal were flagged 'absent' too. Data from spots that not passed this criterion for one channel but with >95% of the pixels >2 SD above median local background signal in the other channel were kept. GenePix result files, including signal, background, standard deviation, pixel statistics and quality parameters on both channels have been imported in the statistical environment R [22] using Bioconductor software [23] for the subsequent normalization process. Background-subtracted fluorescence log-ratios were normalized within each array by using composite loess normalization [14] available in the Bioconductor package limma [23,24]. Composite loess normalization corrects the expression log-ratios for intensity-based trends subtracting from each expression log-ratio the corresponding value of the loess curve. The loess curve is constructed by performing a series of local regressions, one local regression for each spike-in control spot on the corresponding MA-plot [13]. Being R and G, the background-corrected red and green intensities for each spot, the expression log-ratio (M-value) corresponding to a spot is M = log_2_R - log_2_G, whereas the log-intensity (A-value) of each spot is defined as A = (log_2_R+log_2_G)/2, a measure of the overall brightness of the spot. All spike-in control spots (after filtering) have been included in each local estimate of the loess curve (this corresponds to set the parameter span equal to 1 in the implementation of the loess normalization algorithm in the package limma) to avoid a non-reliable representation of the overall trend within the sliding windows used for local regressions due to the low number of genes spotted on the array. Median percentile normalization was performed utilizing the "normalize to median or percentile" option in GeneSpring GX 7.3 (Silicon Genetics, Redwood City, CA).

In some cases, loess normalized M-values have also been scaled across a series of arrays. The need for scaling across arrays has been determined empirically in each instance, according to the experimental evidences on different classes of spots (basically, spike and non-spike genes). We used two different methods for scaling, both implemented in the package limma: the *scale *method [3,5,14], whose basic idea is simply to scale the M-values to have the same median-absolute-deviation (MAD) across arrays; and the *quantile *method [19], which ensures that the M-values have the same empirical distribution across arrays and across channels.

### Analysis of variance (ANOVA)

Analysis of variance (ANOVA) is a procedure for constructing statistical tests by partitioning the total variance into different sources. ANOVA model consists in a separation of a complex variance term into its components [25]. We create a fixed effect model with interaction terms to evaluate the main effects of the potential sources of variance. To confirm the loess normalization we performed ANOVA among data before and after the normalization process. The ANOVA model is:

logR(g, d, s) = μ+G(g)+D(d)+S(s)+GD(gd)+ ε(g, d, s)

where log R(gds) is the measured log ratio for spike g, concentration d, and array position s; μ is the average log ratio over the whole array, G(g) is main effect for spike characteristics, D(d) is the main effect for the spike RNA amount (concentration), S(s) is main effect for position on the array, GD(gd) is a term accounting for effects of the interaction between the spike characteristics and the concentration and ε(g, d, s) is stochastic error. The error is assumed to be independent and of zero mean. To satisfy these assumptions, the homogeneity of the variances was visually inspected by residual graphic analysis. Statistical analysis was performed with SPSS 13.0 (SSPS Inc., Chicago, IL).

### ROC analysis

System specificity and sensitivity in detecting differential gene expression were evaluated using receiver operating characteristic (ROC) curves [26]. A ROC curve shows the relationship between the proportion of true positive (Sensitivity) and false positive (1-Specificity) classifications resulting from each possible decision threshold value in a two-class classification task [20]. The area under the curve is a measure of test accuracy [27], and when applied to a gene expression profile, it provides an estimate of the probability that a gene is up- or down-regulated in a given group. The spike RNAs were added to the hybridization mixture of the arrays at pre-determined specific concentrations ranging from 500 to 1500 pg. Test sensitivity was calculated as the number of regulated genes correctly classified by the test divided by the number of regulated genes. False-positive rate is defined as the number of false positives genes from the group of non-regulated genes divided by the total number of non-regulated genes. Statistical analysis was performed with STATA 8.0 (StataCorp LP, College Station, TX).

### Description of experiments

All microarray raw data were provided as additional files. We performed three types of microarray experiments. (i) In dilution experiments all the spike RNAs were added in the same quantity in both channels. We set up four different dilutions: 10 pg (additional file 1), 250 pg, 750 pg (additional file 2) and 1000 pg (additional file 3). The experiment at 250 pg was performed in quadruplicate (additional files 4, 5, 6, 7). Data from these dilution experiments were used for Figure 1, Figure 2, Figure 3, Figure 4, Figure 7, Figure 8, Table 2, Table 3, Table 4 and Table 5. (ii) In range experiments two different mixtures were set up to cover a wide range of signal intensity. Every spike RNA was added in the same quantity in both channels to get a final ratio of 1, but in the same mixture the spikes were present at increasing concentrations. Mix 1 contains spikes at 5 pg, 10 pg, 50 pg, 100 pg, 500 pg and 1000 pg (additional file 8). Mix 2 contains spikes at 250 pg, 500 pg, 1000 pg, 1500 pg, 3000 pg and 5000 pg (additional file 9). Data from range experiments were used for data in Figure 5. (iii) ROC experiments were planned to compare expected with measured signal ratios. The spike RNAs were added in defined quantity to obtain ratios of 1 (500/500 pg), 1.5 (750/500 pg), 2 (1000/500 pg) and 3 (1500/500 pg) (additional file 10). Dye swap was performed to get reverse ratios (additional file 11). Data from ROC experiments were used in Figure 6.

## Authors' contributions

LV and PF conceived the initial idea, the experimental design, supervised the work, and wrote the manuscript. SM contributed with the development of R scripts for data processing, data normalization and for the identification of differentially expressed genes. BB performed ANOVA and ROC analysis. AR helped with the RNA extraction and microarray experiments. SB contributed to the experimental design and to the writing of the manuscript. All authors read and approved the final manuscript.

## Supplementary Material

Additional File 1 Microarray raw data of the10 pg dilution experiment. All the spike RNAs were added at the same quantity in both channels. Click here for file

Additional File 2 Microarray raw data of the 750 pg dilution experiment. All the spike RNAs were added at the same quantity in both channels. Click here for file

Additional File 3 Microarray raw data of the1000 pg dilution experiment. All the spike RNAs were added at the same quantity in both channels. Click here for file

Additional File 4 Microarray raw data of the 250 pg dilution experiment, replicate A. All the spike RNAs were added at the same quantity in both channels. Click here for file

Additional File 5 Microarray raw data of the 250 pg dilution experiment, replicate B. All the spike RNAs were added at the same quantity in both channels. Click here for file

Additional File 6 Microarray raw data of the 250 pg dilution experiment, replicate C. All the spike RNAs were added at the same quantity in both channels. Click here for file

Additional File 7 Microarray raw data of the 250 pg dilution experiment, replicate D. All the spike RNAs were added at the same quantity in both channels. Click here for file

Additional File 8 Microarray raw data of the mix 1 range experiment. Every spike RNA was added in the same quantity in both channels to get a final ratio of 1, but in the same mixture the spikes were present at increasing concentrations: sp1: 1000 pg; sp2: 500 pg; sp3: 5 pg; sp4: 10 pg; sp5: 1000 pg; sp6: 500 pg: sp7: 50 pg; sp8: 100 pg. Click here for file

Additional File 9 Microarray raw data of the mix 1 range experiment. Every spike RNA was added in the same quantity in both channels to get a final ratio of 1, but in the same mixture the spikes were present at increasing concentrations: sp1: 1500 pg; sp2: 1500 pg; sp3: 250 pg; sp4: 250 pg; sp5: 5000 pg; sp6: 3000 pg: sp7: 500 pg; sp8: 1000 pg. Click here for file

Additional File 10 Microarray raw data of the ROC curve experiment. The spike RNAs were added in defined quantity to obtain ratios of 1 (500/500 pg): sp1, sp4, sp6, sp7; of 1.5 (750/500 pg): sp5; of 2 (1000/500 pg): sp2, sp3; of 3 (1500/500 pg): sp8. Click here for file

Additional File 11 Microarray raw data of the ROC curve experiment. The spike RNAs were added in defined quantity to obtain ratios of 1 (500/500 pg): sp1, sp4, sp6, sp7; of 1.5 (750/500 pg): sp5; of 2 (1000/500 pg): sp2, sp3; of 3 (1500/500 pg): sp8. Dye swap was performed to get reverse ratios. Click here for file

## Figures and Tables

**Table 1:** RNA spikes: length and base composition

RNA spike	Length (nt)	Base Composition^a)^			
		C	U	A	G
sp 1	750	23.5%	26.5%	21.9%	28.1%
sp 2	752	29.7%	23.5%	20.6%	26.2%
sp 3	1000	23.2%	25.9%	16.6%	34.3%
sp 4	1000	23.5%	22.0%	27.0%	27.5%
sp 5	1034	27.5%	22.3%	22.8%	26.4%
sp 6	1250	27.7%	21.8%	22.3%	28.2%
sp 7	1474	27.9%	20.5%	24.4%	27.3%
sp 8	2000	27.2%	21.4%	23.7%	27.8%

^a) ^Expressed as percentage of the base with respect to the total nucleotide content

**Table 2:** Reproducibility of replicate spots within array

Spike	SD^a)^	CV^b)^
sp 1	0.08	5.15%
sp 2	0.05	5.57%
sp 3	0.08	6.91%
sp 4	0.07	6.40%
sp 5	0.11	6.25%
sp 6	0.06	5.50%
sp 7	0.07	7.13%
sp 8	0.08	8.10%

^a)^Standard deviation (SD) of M-values (M = log_2_R-log_2_G) was calculated for each series of 48 replicate spots within array. The averages of the SD of seven independent microarray experiments are shown. ^b) ^Coefficient of variation (CV) [(SD of M-values × 100)/mean of M-values] was calculated on the same sets of data and the averages of the seven independent microarray experiments are shown.

**Table 3:** ANOVA analysis before and after normalization

	Before normalization				After normalization			
	
Source of variation^a)^	sum of squares	degree of freedom	p-value	% of variance	sum of squares	degree of freedom	p-value	% of variance
Spike concentration^b)^	169.084	3	0.000	48.3%	0.762	3	0.000	1.0%
Spike characteristics^c)^	62.295	7	0.000	17.8%	50.094	7	0.000	40.4%
Array position^d)^	1.530	11	0.000	0.4%	1.247	11	0.000	0.6%
Concentration* characteristics^e)^	104.040	21	0.000	29.7%	58.848	21	0.000	47.4%
Error^f)^	13.477	1240		3.8%	13.105	1240		10.6%
Total^g)^	332.992	1282			119.280	1282		

	R Squared^h) ^= 0.958				R Squared^h) ^= 0.887			

^a) ^The variability is subdivided into independent variances. ^b) ^Amount of spikes in the experiment. ^c) ^Base composition, length of the mRNA and interaction with other RNA molecules. ^d) ^Spot size, tips, slide homogeneity, printer accuracy. ^e) ^Interaction between spike concentration and spike characteristics. ^f) ^Residual variability. ^g) ^Corrected total variability. ^h) ^R squared calculated from linearity test resulting from the amount of variance in the dependent variable that is accounted for by the corrected model.

**Table 4:** Variability of spike after excluding outlier values

	Before exclusion	After exclusion	
		
Spike	CV ^a)^	CV ^b)^	Excluded ^c)^
sp1	5.15%	4.85%	14% (1/7)
sp2	5.57%	5.57%	0
sp3	6.91%	6.31%	14% (1/7)
sp4	6.40%	6.40%	0
sp5	6.25%	4.80%	29% (2/7)
sp6	5.50%	5.50%	0
sp7	7.13%	6.48%	14% (1/7)
sp8	8.10%	6.47%	29% (2/7)

^a)^Coefficient of variation (CV) [(SD of M-values × 100)/mean of M-values] was calculated for each series of 48 replicate spots within array before the outliers exclusion. The averages of the seven independent microarray experiments are shown. ^b) ^Coefficient of variation (CV) after the exclusion procedure. The CV was calculated as the mean of the remaining data after the filter. ^c) ^Percentage of experiments in which the single spike was excluded on a total of seven experiments. The number of experiments in which the specific spike was excluded out of the seven arrays analyzed is shown in parenthesis.

**Table 5:** list of up-regulated genes

Gene name	GeneBank^a)^	Description	Fold change^b)^	References^c)^
Vegfa	NM_009505	Vascular endothelial growth factor A	3.66	[11]
Selenbp1	NM_009150	Selenium binding protein 1	2.98	unpublished
Gna13	NM_010303	Guanine nucleotide binding protein, alpha 13	2.92	unpublished
Anxa4	NM_013471	Annexin A4	2.89	[11]
Bsg	AK002332	Basigin	2.62	[28]
F2r	NM_010169	Coagulation factor II (thrombin) receptor	2.60	
P4hb	X06453	Prolyl 4-hydroxylase, beta polypeptide	2.23	[28]
G3pdh	NM_008084	Glyceraldehyde-3-phosphate dehydrogenase	2.07	[29]
Bnip3l	NM_009761	BCL2/adenovirus E1B interact protein 3-like	2.00	[30]
Foxg1	NM_008241	Forkhead box G1	1.97	
Ifi204	NM_008329	Interferon-inducible protein p204	1.96	
Ctsd	NM_009983	Cathepsin D	1.95	[31]
Btg1	L16846	B-cell translocation gene 1	1.93	[32]
Es10	NM_016903	Esterase 10	1.83	unpublished
Ecm1	NM_007899	Extracellular matrix protein 1	1.79	unpublished
Cxcr4	AB000803	Chemokine (C-X-C motif) receptor 4	1.77	[33]
Il15	NM_008357	Interleukin 15	1.76	unpublished

^a) ^GeneBank accession number. ^b) ^The indicated values represent the ratio of hypoxic/normoxic signals (mean of 4 experiments). ^c) ^Genes validated in published literature or by our unpublished observation using independent mRNA assessment methods.
